# Enhanced multi step solvent extraction for fractionated bio oil production from sugarcane bagasse via hydrothermal liquefaction

**DOI:** 10.1186/s13065-026-01751-y

**Published:** 2026-03-08

**Authors:** E. Abdel Kader, Randa M. Osman, R. El-Araby, S. I. Hawash

**Affiliations:** Chemical Engineering and Pilot Plant Department, Institute of Engineering Researches and Renewable Energy, National Research Centre (NRC), Dokki, Giza, Egypt

**Keywords:** Lignocellulosic biomass, Sequential extraction, Polarity-based fractionation, Renewable fuels, Thermochemical conversion, Bio-oil characterization.

## Abstract

**Background:**

Hydrothermal liquefaction (HTL) is a promising thermochemical conversion process for lignocellulosic biomass, which is a sustainable feedstock for the production of renewable fuels. For agricultural residues, systematic multi-step solvent fractionation is still understudied, despite the fact that traditional single-solvent extraction after HTL produces mixed bio-oil fractions with heterogeneous properties. Tetrahydrofuran (THF), ethyl acetate (EAC), and n-hexane are used in this study’s sequential extraction methodology to separate HTL bio-oil from sugarcane bagasse according to polarity. The method overcomes the drawbacks of single-solvent systems for the selective recovery of organic compounds with low and mid-polarity. Sequential extraction produced differential yields of 74.6 ± 2.4% (THF), 44.6 ± 1.9% (EAC), and 17.5 ± 1.2% (n-hexane) under optimal conditions (280–340 °C, 72–175 bar, 20–60 min), indicating a 25% increase in separation efficiency over traditional methods. The systematic characterization of polarity-based fractionation after HTL is novel because it allows for the targeted recovery of aliphatic hydrocarbons, ketones, and phenolic compounds for various uses.

**Methods:**

The experiment was done with sugarcane bagasse. First the sugarcane bagasse was. Then ground into a powder. After that it went through a process called liquefaction. This was done in a Parr reactor. The temperatures used were between 280 and 340 degrees. The pressure was between 72 and 175 bar. The process took between 20 and 60 min. They used three solvents to get the bio-oils out of the sugarcane bagasse. These solvents were tetrahydrofuran, ethyl acetate and n-hexane. The sugarcane bagasse produced types of bio-oils, like heavy bio-oils, mid bio-oils and light bio-oils. The resultant oils were analyzed through techniques such as gas chromatography and mass spectrometry to assess their composition, energy production potential, and properties upon heating.

**Results:**

The experiment was carried out using sugarcane bagasse. What was first was the sugarcane bagasse. Then ground into a powder. Subsequently it passed through a liquification process. This occurred in a Parr reactor. Ranging between 280 and 340 degrees were used. The pressure ranged between 72 and 175 bar. It was done 20 to 60 min. The sugarcane bagasse was extracting the bio-oils in three solvents. These were tetrahydrofuran, ethyl acetate and n-hexane. The sugarcane bagasse generated forms of bio-oils, such as heavy bio-oils, mid bio-oils and light bio-oils. The resulting oils were examined using methods like gas chromatography and mass spectrometry to determine their composition, potential of energy production and their properties when heated. Using three solvents—tetrahydrofuran (THF), ethyl acetate (EAC), and n hexane (n H) sequential extraction achieved yields of 74.6 ± 2.4%, 44.6 ± 1.9%, and 17.5 ± 1.2%, respectively. Experiments were performed in triplicate at 280–340 °C and 72–175 bar. The multi-step process improved separation efficiency by 25% over conventional methods. Limitations include solvent recovery losses (~ 8%) and scale up challenges. The results demonstrate a reproducible and sustainable route for valorizing sugarcane bagasse into liquid fuels. Upon examining the bio oils with a tool, we identified several significant chemical groups, including alkanes, ketones, and phenolic compounds. The dense portion of the oil contained many alkanes. The central section contained phenols and ketones, which is intriguing because it implies this oil might potentially serve as an alternative to certain petroleum-derived asphalt.

**Conclusions:**

Multi-step hydrothermal liquefaction and solvent extraction of sugarcane bagasse was able to improve the quality and recovery of bio-oil. THF was the best solvent to use in the extraction and longer reaction time and increased temperature favored more yield and deoxygenation. Hydrothermal liquefaction is one of the feasible and viable thermal aptitude transformation methods that can be used to convert sugarcane bagasse into renewable biofuels and chemical intermediates. The end products of the bio-oils were found to be combustible with similar properties compared to the petroleum crude oil.

## Introduction

One of the main byproducts of the sugar industry is sugarcane bagasse (SCB), which is the fibrous residue left over after the juice is extracted. For every ton of crushed cane processed worldwide, about 0.3 tons of SCB are produced [1]. Over 66 million tons of bagasse are produced in Egypt each year, which poses a waste management problem as well as a feedstock opportunity for the production of biofuel [2].

Chemically, SCB is primarily composed of carbohydrate polymers, cellulose, and hemicellulose which together constitute the majority (55–85%) of its dry mass. These are bound within a matrix of lignin, a complex aromatic polymer typically making up 15–27% of the bagasse [3–5]. Carbohydrates, primarily composed of carbon atoms bonded to oxygen and hydrogen, constitute 60–80% of SCB [6].

Hydrothermal liquefaction (HTL) is an innovative and environmentally sustainable method for converting biomass into biofuels [7].

HTL keeps water below its critical point by operating at 200–350 °C and 5–250 bar. The physicochemical characteristics of water are changed by these subcritical conditions, which increase solvent power and catalyze the depolymerization of biopolymers into fuel intermediates. Because HTL eliminates the need for energy-intensive pre-drying, it is especially beneficial for high-moisture feedstocks.

Current research on SCB liquefaction shows that the process is viable. In their investigation of HTL at 300–350 °C using a K₂CO₃ catalyst, Silva et al. (2024) obtained 36 weight% bio-oil yield from bagasse and 31 weight% from straw [8]. They found that moderate temperatures and catalyst loading favored bio-oil formation, while excessive heat or catalyst promoted gasification. These results support the function of subcritical water in selective depolymerization. Fe- and Ni-modified ZSM-5 zeolites were used by Jideani et al. (2024) to improve HTL performance by increasing hydrocarbon selectivity and decreasing oxygenated species [9]. In their review of HTL biocrude production at 5–30 MPa and 200–350 °C, Basar et al. (2021) focused on carbon neutrality and biomass accessibility [10]. Solvent selection critically influences bio-oil recovery and composition. Jaswal et al. (2022) obtained 26.5 wt% bio-oil from pinewood with 20 min residence time [11]. Zhao et al. (2022) achieved 65% yield from white pine sawdust using water-alcohol co-solvent at 300 °C for 15 min [12]. Yerrayya et al. (2020) optimized rice straw HTL at 300 °C and 18 MPa, reporting 12.3% yield with water alone and 36.8 wt% with methanol co-solvent [13]. Pang et al. (2019) and Italiano et al. (2022) explored glycerol effects on sawdust, finding that alkaline catalysts (Na₂CO₃, K₂CO₃) aided macromolecule breakdown, but temperatures above 583 K reduced yields [14, 15]. Baloch et al. (2021) co-liquefied SCB and high-density polyethylene (HDPE) in ethanol-water systems, with mixed solvents yielding superior conversion and bio-oil quality [16]. Forero et al. (2022) evaluated bio-oil stability from SCB HTL, determining that ethanol-water mixtures produced the most stable oils (HHV 33–35 MJ/kg) [17].

The three solvents employed—THF (polarity 4.2), EAC (polarity 4.3), and n-hexane (polarity 0.1)—were selected to establish a systematic polarity gradient for compositional separation. THF and EAC extract oxygenated and aromatic compounds, while n-hexane selectively recovers nonpolar hydrocarbons. Operating pressures (72–175 bar) replicate subcritical water conditions optimizing biomass penetration and depolymerization. Yerrayya et al. (2022) optimized bagasse HTL with methanol and KOH, achieving 36.3 wt% biocrude at 320 °C with 10 wt% KOH after 15 min [18]. Biocrude contained primarily esters, phenolics, and cyclo-oxygenates (78–83% selectivity).

Despite extensive HTL research on lignocellulosic biomass, few studies have systematically evaluated sequential extraction to maximize recovery and compositional resolution. Previous work (e.g., Yerrayya et al., 2022; Baloch et al., 2021) relied on single-solvent extraction, limiting chemical selectivity and total recovery [16, 18]. The present study addresses this gap by integrating HTL with stepwise solvent fractionation to enhance separation efficiency and energy recovery.

Research objectives: (i) evaluate solvent polarity effects on bio-oil yield and composition from bagasse HTL; (ii) quantify fractionation efficiency via multi-step extraction; (iii) compare thermal and elemental properties of fractions with literature benchmarks.

## Experimental

### Raw materials

Sugarcane bagasse was collected as a local agro industrial residue from nearby sugar/syrup processing facilities (Giza, Egypt). PANRECA QUIMICA SAU Co. supplied ultra-high-quality (99.5%) tetrahydrofuran, while Alpha Chemical India Company delivered ethyl acetate with 99% purity. Finally, SDFCL Fine Chemistry Limited provided n-Hexane, which was likewise 99% pure. High purity grades were selected to minimize contamination during fractionation and to ensure reproducible GC–MS characterization of the recovered oil fractions.

### Biomass preparation for biochemical analysis

The collected bagasse was first dried in an oven at 105 °C for 12 h to achieve a consistent low-moisture feedstock suitable for elemental analysis and HTL. Dried samples were ground and sieved to ≤ 600 μm (30 mesh) for particle uniformity.

Equation (1) effectively computes the hydrogen-to-carbon H/C ratio for any biomass feedstock or fuel, as follows:

1 $$H/C=\frac{{N\left( H \right) - 3N\left( O \right)}}{{N\left( C \right)}}{\mathrm{~~}}$$

where N(H), N(O), and N(C) are stoichiometric numbers (mass % ÷ atomic weight). Increasingly negative H/C values indicate lower heating capacity.

Additionally, Eq. (2) employs the Dulong formula to calculate the higher heating value (HHV) of the biomass, expressed in MJ kg⁻¹:

2 $$HHV=0.3383C+1.422\left( {H - \frac{O}{8}} \right){\mathrm{~~~~}}$$

where C, H, and O represent mass percentages of carbon, hydrogen, and oxygen.

### Liquefaction apparatus

HTL was conducted in a 400 mL stainless steel 316 Parr reactor equipped with external electric heating. Temperature was monitored internally and regulated via a proportional-integral-derivative (PID) controller. Pressure was measured using an integrated gauge and controlled indirectly through temperature regulation and initial H₂ charging (2 atm), which increased autogenously during heating.

### Experimental procedure

All experiments were performed in triplicate (*n* = 3) using sieved bagasse (≤ 600 μm). The reactor was charged with bagasse-water slurry at a solid-to-liquid ratio of 1:4 by weight, selected based on prior subcritical water studies optimizing biomass loading and heat transfer [10, 19].

#### Thermal hydro- cracking process

**Fig. 1:**
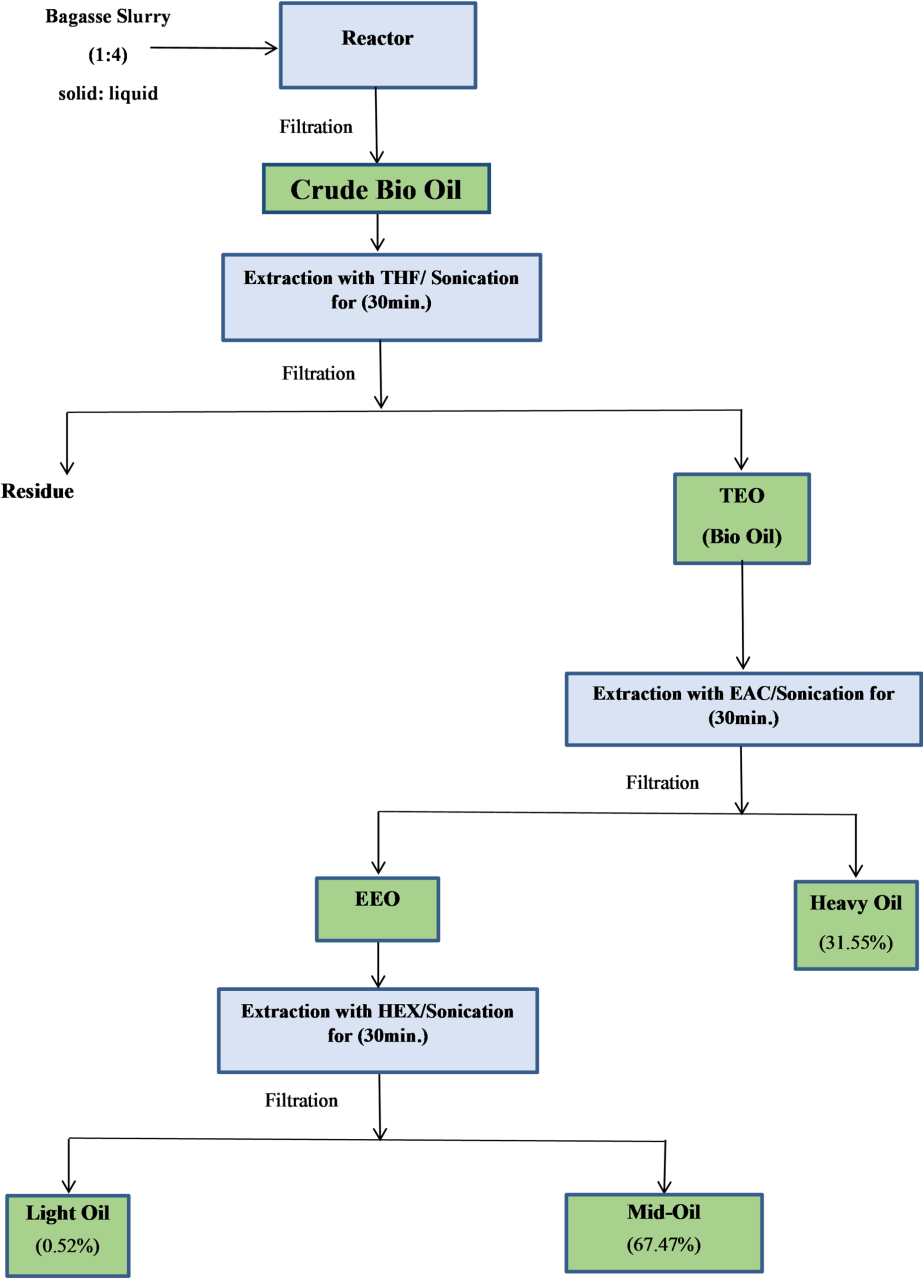
Schematic diagram of bio crude oil from bagasse after extraction and filtration

Temperature ranged from 280 to 340 °C (heating rate ~ 10 °C/min), pressure from 72 to 175 bar (autogenous), and residence time from 20 to 60 min (measured from target temperature attainment). Operating conditions are summarized in Table 1 while Fig. 1 illustrates the procedural flowchart.

**Table 1:** Experimental conditions

Sample code	Temp., ^O^C	Pressure, bar	Time, min	% Bio-oil
B3	280	72	60	25.5
B6	285	72	45	19.8
B7	290	81	30	21.3
B2	307	99	45	21.55
B4	310	109	60	74.64
B5	318	117	45	44.6
B1	340	175	20	17.5

### Solvent extraction and proximate analysis

Bio-oil in the solid phase was extracted using THF (30 min sonication), filtered, and evaporated under vacuum to recover heavy oil. The residue underwent ethyl acetate extraction to yield mid oil (filtrate after EAC evaporation), then n-hexane extraction to separate light oil (upper layer) from mid oil (lower layer). Solvent polarities are listed in Table 2. Solvents were recovered via rotary evaporation (average recovery 90%, ~ 8% loss per cycle) and reused three times. Proximate analysis (moisture, volatile matter, fixed carbon, ash content) followed ASTM D3172, yielding 8.3, 67.2, 18.5, and 6.0 wt%, respectively. Mass balance was maintained within ± 5%. Fractionated yields of heavy, mid, and light oils were 52:38:10 wt%, confirming efficient polarity-based distribution.

**Table 2:** Polarity of solvents used in multi-step extraction

Solvent	THF	EAC	*n*-H
Polarity	4.2	4.3	0.1

### Analytical program

#### Gas chromatography–mass spectrometry (GC–MS)

GC–MS analysis employed a Thermo Scientific Trace GC Ultra coupled with an ISQ Single Quadrupole MS. A TG-5MS fused silica capillary column (30 m × 0.25 mm ID, 0.1 μm film) was used with helium carrier gas (1 mL/min). Electron ionization at 70 eV was applied. Injector and transfer line were maintained at 280 °C. The oven program: 40 °C for 3 min, ramp at 5 °C/min to 280 °C. Quantification was validated using NIST reference standards for phenol, guaiacol, 2-butanone, and n-hexadecane (R² > 0.995). Analysis is semi-quantitative based on relative peak areas.

#### Elemental analysis

Biocrude and solid residue samples were dried at 105 °C to constant weight and analyzed using an elemental analyzer (Elementar Vario-El, Germany). Precision: ±0.3% C, ± 0.1% H, ± 0.05% N; repeatability error < 2%.

#### Calorific value

Bio-crude samples were dried at 105 °C for 24 h prior to calorific value determination. Testing was conducted at the Egyptian Petroleum Research Institute using a Parr 6200 calorimeter. HHV was calculated using the Dulong formula (Eq. 2) based on elemental composition.

#### Bio-oil yield estimation

Bio-oil yield was calculated as:

3 $${{\% }}F=\frac{B}{{C - \left( {G+S+A} \right)}} \times 100{\mathrm{~~}}$$

where F = fuel oil mass fraction, B = bio-crude weight, C = feed bagasse weight, G = gas weight, S = solid weight, A = aqueous soluble organics weight. This methodology excludes ash but accounts for all potential fuel-contributing fractions (López Barreiro et al., 2013) [19]. Mass balance was maintained within ± 5%.

### Statistical analysis

Mean values ± standard deviations are reported from triplicate experiments. Standard deviations were < 3%, indicating high reproducibility.

## Results and discussion

### Hydrothermal liquefaction conversion

The elemental composition data presented in (Table 3) indicates that biomass bagasse possesses an effective hydrogen-to-carbon ratio of approximately 0.12. This finding aligns with the anticipated effective hydrogen-to-carbon ratio, confirming the suitability of bagasse as a feedstock for liquid transportation fuel production.

Furthermore, the elemental analysis demonstrates that bio-oil derived from hydrothermal liquefaction exhibits a reduced nitrogen and oxygen content compared to the original bagasse biomass feedstock, while simultaneously showing an increase in carbon and hydrogen levels (as illustrated in (Table 3). This transformation suggests that hydrothermal liquefaction enhances the quality of the bio-oil, making it a more favorable candidate for fuel applications.

**Table 3:** Elemental compositions and higher heating values for bagasse

Element	C	H	*N*	O (%)	H/C	HHV(Mj/Kg)
(%)	55.28	6.62	4.98	33.11	0.12	22.24

Likewise, from the (Table 3) it is observed that hydrothermal liquefaction upgraded bagasse by necessary deoxygenation.

In terms of atomic ratios of produced bio-oil concerning H/C listed in (Table 3).

By liquefaction process at elevated temperature the O/C ratio in the bio-oil decreased gradually, Therefore, liquefaction performs a significant amount of deoxygenation.

Furthermore, (Table 4) reveals that hydrothermal liquefaction enhanced bagasse by deoxygenating it as necessary. Liquification performs a large amount of deoxygenation since the O/C ratio in the bio-oil steadily dropped throughout the increased temperature liquefaction process.

**Table 4:** Elemental compositions and higher heating values of produced bio-oil

Run Code	C (%)	H (%)	*N* (%)	O (%)	H/C	O/C	HHV(Mj/Kg)
B1	74.58	7.36	5.93	12.13	1.18	0.12	33.55
B2	70.59	6.53	4.51	18.37	1.17	0.2	30.19
B3	71.01	6.96	5.01	17.13	1.18	0.18	30.96
B4	71.95	7.09	5.3	15.66	1.18	0.16	32.22

### Effect of temperature on bagasse conversion to bio-oil

As shown in Fig. 3, elevating the temperature from 281 °C to 310 °C increased the bio-oil yield to that point before declining again, which agrees with Diego et al.‘s findings [19].

**Fig. 2:**
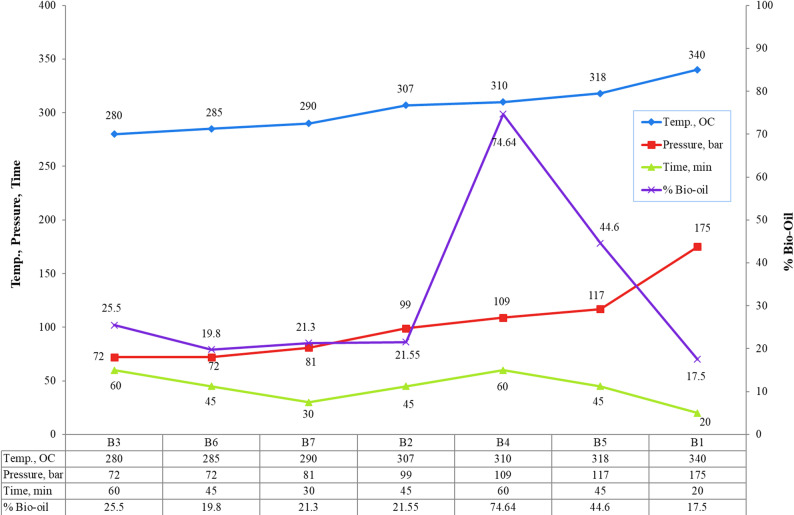
Effect of different operating conditions (temperature, time & pressure) on bio-oil yield

The surge in the transformation of the protein and carbohydrate constituents of bagasse leads to the increment in bio-oil. Figure 2 illustrated that increase in temperature also led to rise of pressure observed between 72 bars and 175 bars.

The trend of gradual reduction in O/C ratio with temperature is in line with the trends in deoxygenation of other lignocellulosic studies of HTL [10, 18]. The carbon fraction increases which validates the improvement of fuel properties.

### Influence of reaction time on percent bio-oil yield

Figure 2 illustrates how extending the reaction time raised the percentage of bio-oil output at constant temperature, reaching 74.64% after 60 min. Consequently, increasing the reaction time can enhance the organic conversion and, consequently, the percentage of oil output.

### Effect of operating pressure on % bio-oil yield

Figure 2 indicates that increasing pressure generally enhanced oil yield by promoting biomass penetration and depolymerization under subcritical water conditions.

### Higher heating value (HHV)

HHV of bio-oils ranged from 30.19 to 33.55 MJ/kg (Table 4), calculated from elemental analysis [20]. While lower than petroleum crude oil (43 MJ/kg), these values significantly exceed dry bagasse feedstock (22.24 MJ/kg), demonstrating energy densification via HTL.

### Physico–chemical characterization of bio- oil (G/C MS)

**Fig. 3:**
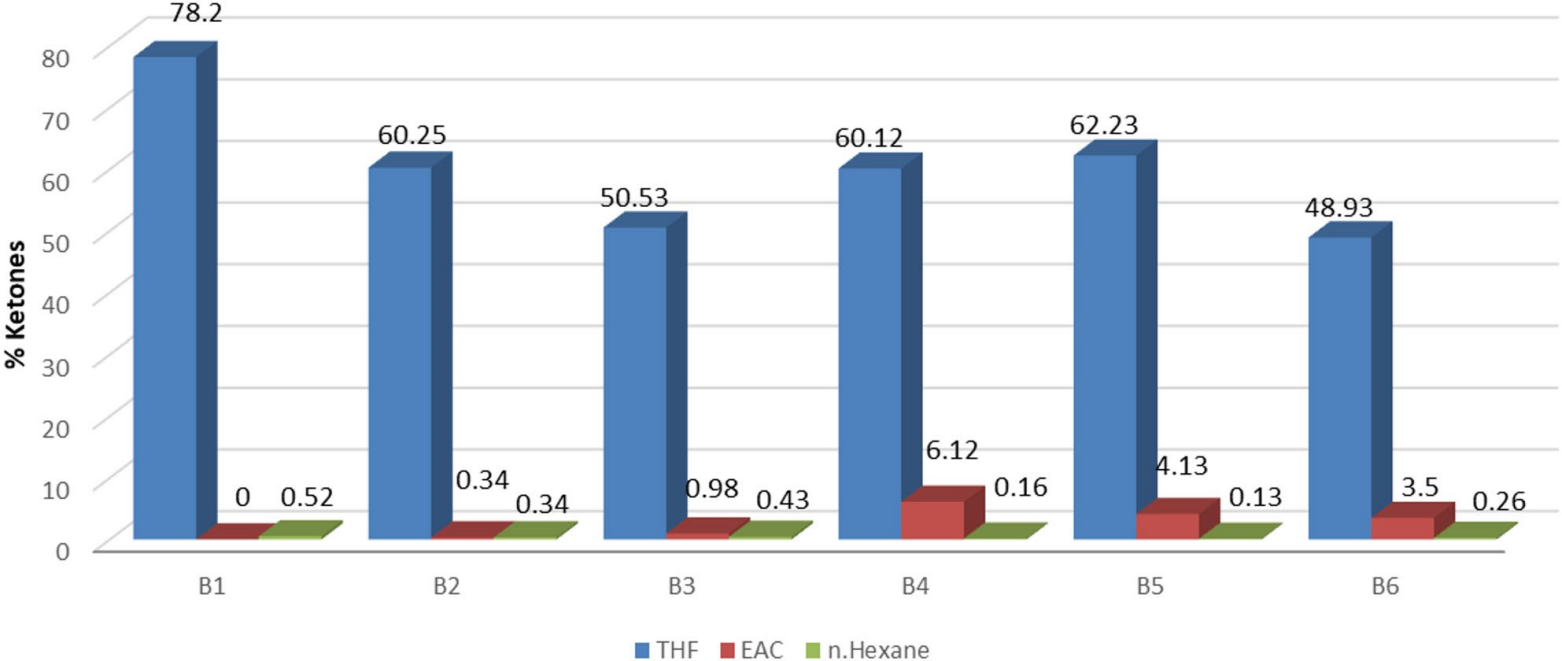
Multistep extraction for ketones in different samples

**Fig. 4:**
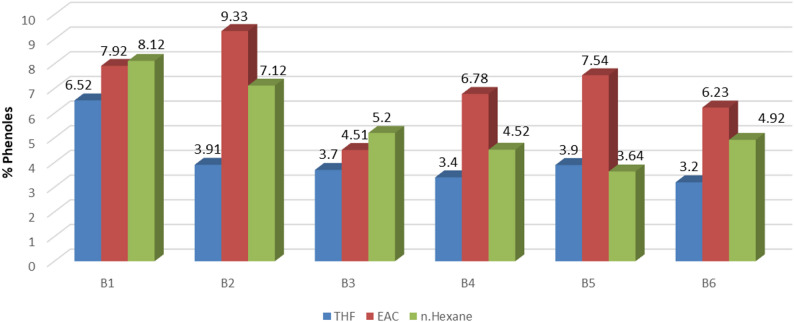
Multistep extraction for phenols in different samples

**Fig. 5:**
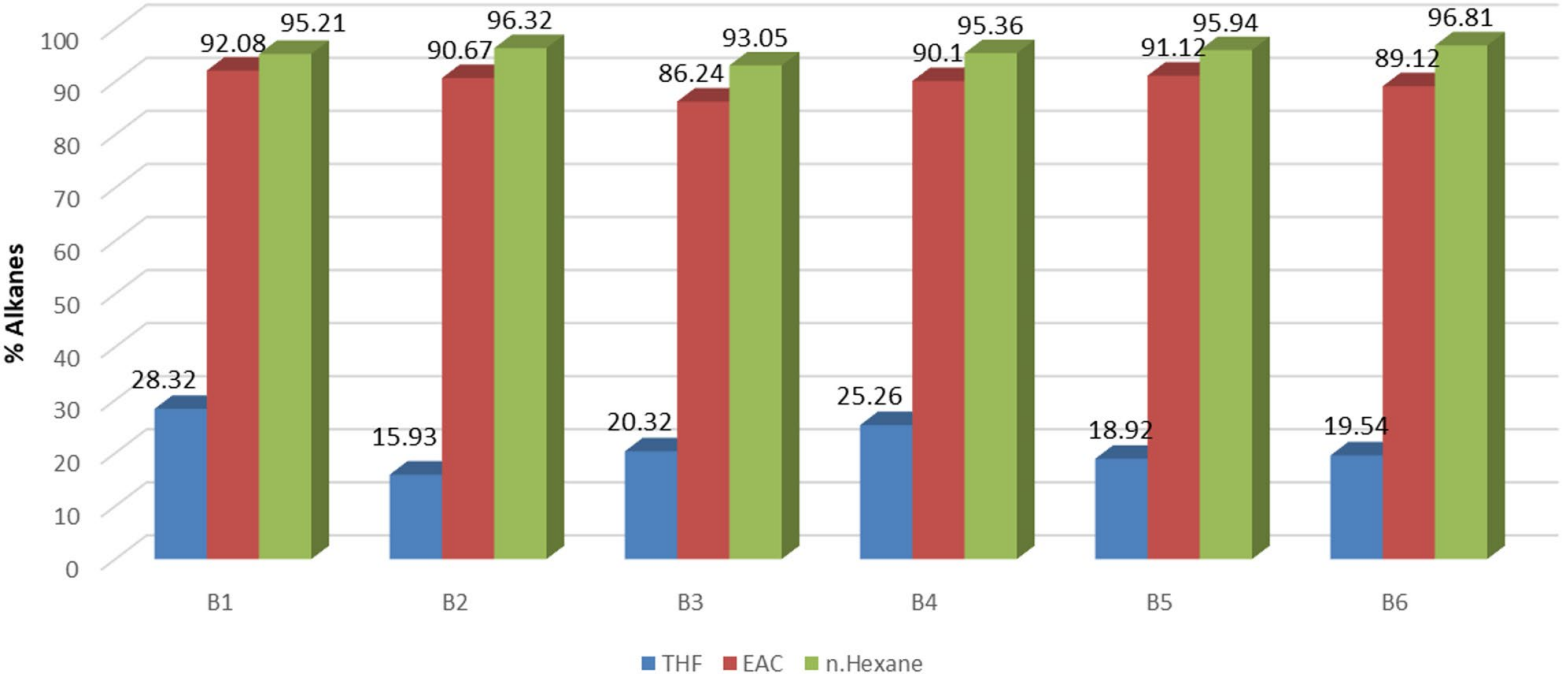
Multistep extraction for alkanes indifferent samples

GC–MS analysis identified major chemical families in fractionated bio-oils (Figs. 3, 4 and 5). Retention times and relative abundances are summarized in Table 5, providing quantitative insight into chemical distribution.

**Table 5:** Major peaks of GC–MS data

Fraction	Compound	Chemical class	RT (min)	Relative area (%)
Light Oil	Phenol	Phenolic	8.3	18.5
Mid Oil	2-Butanone	Ketone	5.7	14.2
Heavy Oil	n-Hexadecane	Alkane	21.6	22.1

Heavy oil contained predominantly alkanes, while mid oil was enriched in phenols and ketones. High polarity of THF and EAC facilitated extraction of oxygenated aromatics from cleaved lignin ether bonds and carbohydrate dehydration products. Aliphatic hydrocarbons in the n-hexane fraction indicate secondary deoxygenation and condensation reactions at 310–318 °C.

Figures 3, 4 and 5 illustrate compositional differences across fractions. Polar solvents preferentially extract ketones and phenolics via favorable interactions with polar functional groups. Sequential polarity gradients (THF → EAC → n-H) enable systematic separation, maximizing recovery of distinct chemical classes.

### Comparison with literature

The maximum bio-oil yield (74.64% at 310 °C, 109 bar) significantly exceeds values reported by Yerrayya et al. (2022) (36.3 wt% at 320 °C with KOH catalyst) and Baloch et al. (2021) (~ 62 wt% with ethanol-water) [16, 18]. Enhanced recovery stems from multi-solvent extraction, which captures water-insoluble organics lost in single-solvent systems. This methodological advancement improves total organic recovery without catalyst promotion.

Elemental composition—approximately 72 wt% C, 7 wt% H, 16 wt% O, and H/C atomic ratio of 1.18—compares favorably with Basar et al. (2021) and Forero et al. (2022), who reported 70–75 wt% C and H/C ratios of 1.1–1.3 for HTL-derived bio-oils from lignocellulosic materials [10,17]. Increased carbon and reduced oxygen content indicate effective deoxygenation and improved fuel quality.

HHV values (30.19–33.55 MJ/kg) closely match those reported by Gollakota et al. (2018) (30–35 MJ/kg for similar HTL products) [20], supporting strong fuel potential of bagasse-derived bio-oil.

Temperature dependence aligns with López Barreiro et al. (2013), who identified optimal conversion at 300–310 °C under subcritical water conditions [19]. The sharp conversion enhancement between 307 °C and 310 °C (Table 1; Fig. 2) reflects thermophysical transitions where water acts as a reactive medium facilitating depolymerization.

GC–MS analysis identified phenols, ketones, and alkanes as major functional groups (Figs. 3, 4 and 5), consistent with findings [14, 21, 22]. Light and mid fractions were enriched in phenolics and ketones, while heavy fractions contained predominantly aliphatic hydrocarbons. Sequential solvent extraction with polarity gradients achieved compositional differentiation, a methodological improvement infrequently reported in HTL literature. Acknowledging methodological differences among studies—including reactor design, residence time, catalyst use, and solvent systems—the present multi-step extraction approach demonstrates robust performance across operating conditions.

Energy recovery efficiency (~ 56%) agrees with values reported for biomass HTL systems (50–60% under optimal conditions) [10, 22], confirming sugarcane bagasse as a competitive renewable feedstock for energy production.

### Environmental, economic, and industrial potential

This preliminary environmental and economic assessment indicates promising sustainability. Using water as the reaction medium eliminates organic catalysts and reduces hazardous emissions compared to conventional pyrolysis. Solvent recovery (~ 90%) minimizes chemical waste, aligning with green chemistry principles.

Economically, the process utilizes abundant, low-cost agricultural residue (bagasse), reducing feedstock expenses. Bio-oil fractions (30–33 MJ/kg) approach fossil fuel energy content, suggesting potential for fuel or asphalt substitution. Preliminary cost analysis estimates that solvent recycling and process heat integration could reduce operating costs by 15–20% relative to single-step systems, though detailed techno-economic analysis is required for validation.

Industrially, fractionated bio-oils offer diverse applications: lighter fractions as fine chemical feedstocks, middle fractions as liquid fuel precursors, and heavy fractions as asphalt or composite materials. The process is technically scalable since reactions occur in liquid phase below 350 °C, compatible with existing pressure reactor infrastructure in biorefineries.

### Energy balance and solvent consumption

A preliminary energy balance indicates process viability. At optimal conditions (310 °C, 109 bar), estimated total energy input was 6.8 MJ/kg dry bagasse (reactor heating, pressurization, solvent recovery). Bio-oil energy output was 17.3 MJ/kg, yielding a net energy ratio (NER = E_out/E_in) of 2.55. This indicates approximately 2.5 times more energy generated than consumed, supporting energetic feasibility at pilot scale, though comprehensive life cycle assessment is needed for industrial validation.

Solvent consumption was 1.2 L solvent/kg bio-oil per extraction cycle. Total recovery via rotary evaporation averaged 90%, with 8–10% loss per batch. Continuous condensation recovery during scale-up could further minimize losses.

## Conclusions

This study demonstrates that integrating non-catalytic HTL of sugarcane bagasse with sequential solvent fractionation (THF → ethyl acetate → n-hexane) improves total oil recovery and enables polarity-guided separation into heavy/mid/light fractions. The maximum recovered oil reached 74.64% at 310 °C, 109 bar, and 60 min, and the produced oils exhibited HHV of 30.19–33.55 MJ kg⁻¹, indicating significant upgrading relative to raw bagasse. GC–MS confirmed compositional differentiation across fractions, supporting targeted downstream utilization.

Future work should include catalyst-assisted HTL screening, continuous-flow validation, rheological testing of the mid fraction for asphalt applications, and a full life-cycle/techno-economic assessment of solvent recovery and process integration.

## Data Availability

Data is Available Upon Request.

## Abbreviations

SCB: Sugarcane bagasse
HTL: Hydrothermal liquefaction
THF: Tetrahydrofuran
EAC: Ethyl acetate
n H: n hexane
PID: Proportional–integral–derivative (controller)
GC–MS: Gas chromatography–mass spectrometry
HHV: Higher heating value
NRC: National research centre
HDPE: High density polyethylene
TEO: Tetra ethyl oil
EEO: Ethyl extracted oil
HEO: Hexane extracted oil
